# HOUSING CONCERNS AND TRAJECTORIES OF KINLESS OLDER ADULTS WITH DEMENTIA

**DOI:** 10.1093/geroni/igad104.2828

**Published:** 2023-12-21

**Authors:** Tam Perry, Janelle Taylor, Yasir Mehmood

**Affiliations:** Wayne State University, Detroit, Michigan, United States; University of Toronto, Toronto, Ontario, Canada; Wayne State University, Detroit, Michigan, United States

## Abstract

Housing preferences of older adults (e.g., to remain in residence, or relocate), housing options available, and housing needs related to changing physical and cognitive abilities are common concerns of aging. The challenges are possibly more complex for those living with dementia, who may have difficulty articulating housing preferences, and whose housing needs may include supportive care. For those with dementia who lack close kin, these challenges are likely significantly compounded. This paper focuses on the intersection of housing concerns for 64 participants in the Adult Changes in Thought (ACT) study who were kinless (no living spouse or children) when they developed dementia. All the ACT participants were 65+ when recruited. Everyone in this sample had received a research diagnosis, and their mean age at dementia onset (estimated at the midpoint between the research study that triggered the diagnostic evaluation, and the last research study before that -- usually one year before diagnosis) was 87 (Standard Deviation [SD] 7 years), with a median age of 86 and a range of 71-103.We present an analysis of their housing trajectories, as captured in chart notes from their medical records. We highlight multiple themes illustrating how housing intersects with health and social well-being and conclude by commenting on the potential of medical records for understanding and documenting issues related to housing.

